# Neuro- and Cardiovascular Activities of *Montivipera bornmuelleri* Snake Venom

**DOI:** 10.3390/biology11060888

**Published:** 2022-06-09

**Authors:** Christina Sahyoun, Wojciech Krezel, César Mattei, Jean-Marc Sabatier, Christian Legros, Ziad Fajloun, Mohamad Rima

**Affiliations:** 1 INSERM, CNRS, MITOVASC, Equipe CarMe, SFR ICAT, University of Angers, 49000 Angers, France; christina.sahyoun1@gmail.com (C.S.); cesar.mattei@univ-angers.fr (C.M.); christian.legros@univ-angers.fr (C.L.); 2 Laboratory of Applied Biotechnology (LBA3B), Azm Center for Research in Biotechnology and Its Applications, EDST, Lebanese University, Tripoli 1300, Lebanon; 3 Institut de Génétique et de Biologie Moléculaire et Cellulaire (IGBMC), INSERM, CNRS, Université de Strasbourg, 67400 Illkirch, France; krezel@igbmc.fr; 4 CNRS, INP, Institute of Neurophysiopathology, Aix-Marseille University, 13385 Marseille, France; 5 Department of Biology, Faculty of Sciences 3, Campus Michel Slayman, Lebanese University, Tripoli 1352, Lebanon

**Keywords:** *Montivipera bornmuelleri*, neurotoxicity, cardiotoxicity, zebrafish

## Abstract

**Simple Summary:**

Snake venoms are rich in molecules acting on different biological systems, and they are responsible for the complications following snake bite envenoming. These bioactive molecules are of interest in pharmaceutical industries as templates for drug design. Different biological activities of *Montivipera bornmuelleri* snake venom have been already studied; however, the venom’s activity on the nervous system has not yet been studied, and its effect on the cardiovascular system needs further investigation. Herein, we show that this venom induces toxicity on the nervous system and disrupts the cardiovascular system, highlighting a broad spectrum of biological activities.

**Abstract:**

The complications following snake bite envenoming are due to the venom’s biological activities, which can act on different systems of the prey. These activities arise from the fact that snake venoms are rich in bioactive molecules, which are also of interest for designing drugs. The venom of *Montivipera bornmuelleri*, known as the Lebanon viper, has been shown to exert antibacterial, anticancer, and immunomodulatory effects. However, the venom’s activity on the nervous system has not yet been studied, and its effect on the cardiovascular system needs further investigation. Because zebrafish is a convenient model to study tissue alterations induced by toxic agents, we challenged it with the venom of *Montivipera bornmuelleri*. We show that this venom leads to developmental toxicity but not teratogenicity in zebrafish embryos. The venom also induces neurotoxic effects and disrupts the zebrafish cardiovascular system, leading to heartbeat rate reduction and hemorrhage. Our findings demonstrate the potential neurotoxicity and cardiotoxicity of *M. bornmuelleri*’s venom, suggesting a multitarget strategy during envenomation.

## 1. Introduction

Although dangerous, snake venoms are associated with healing in traditional medicine. Therefore, researchers have extensively explored snake venom composition and biological activities, which has raised interest of pharmaceutical companies for the use of snake venom in drug discovery [1].

*Montivipera bornmuelleri* is a venomous snake mainly present in the Middle Eastern region (reviewed in [2]). This snake is classified among the family of Viperidae, a family of extensively studied venomous snakes. Its venom composition has previously been studied using different analytical techniques, showing a proteomic profile of more than 60 protein compounds [3]. Among these compounds, serine proteases, phospholipase A2 (PLA2), metalloprotease III, and L-amino acid oxidase (LAAO) have previously been reported [2]. At the functional level, some of these compounds confer to the venom biological activities such as antibacterial [3,4], pro- and anticoagulant [5], hemolytic [5], pro-inflammatory [6], vasorelaxant [7], anticancer [8,9], and immunomodulatory effects [10].

Vipers’ venoms can target the cardiovascular and the nervous system of their prey, among others, resulting in organ-related complications [11]. *M. bornmuelleri*’s venom has been shown to act on the vascular system as a vasorelaxant [7,12]; nevertheless, the effect of *M. bornmuelleri*’s venom on the nervous system has not yet been studied. In addition, the effect of the venom on the cardiovascular system needs further investigation. Therefore, we assessed the toxicity of *M. bornmuelleri*’s venom in vivo using zebrafish embryos as a study model. In addition, we screened for neurotoxic and cardiotoxic activities using functional tests on zebrafish embryos. In fact, by being more complex than cultured cells [13], the zebrafish model offers several benefits for assessing snake venom toxicity. The high degree of genetic, morphological, and physiological homology with humans is one of its main advantages [14,15]. In addition, the rapid development of zebrafish makes it a convenient model to evaluate developmental toxicity [16,17,18]. Therefore, the zebrafish model offers a fast and reliable screening system for toxicants at the cellular, molecular, and behavioral scale. This is facilitated by the permeability of the transparent embryo’s membrane that makes the entry of dissolved compounds (i.e., snake venom) simple and effective through passive diffusion [19,20,21].

The promising strategy using zebrafish as a toxicological model system emerged recently for developmental neurotoxicity screening [22,23,24,25]. In fact, spontaneous side-to-side contractions of zebrafish trunk, consisting of the earliest motor behavior of embryos, reflect the advanced development of sensory-motor neuronal circuits [26,27,28]. Since the developing nervous system is sensitive to chemicals, analysis of zebrafish spontaneous tail coiling activity allowed to screen for neurotoxicants and to differentiate their different modes of action [24]. In addition, the transparency of the embryos grants the evaluation of many physiological aspects by simple microscopic observation such as hemorrhage, blood flow, and cardiology measurements. Herein, we took advantage of the zebrafish embryos to investigate the neurotoxicity of *M. bornmuelleri*’s venom and its effects on the cardiovascular system.

## 2. Materials and Methods

### 2.1. Venom

Lyophilized venom was supplied by Riyad Sadek (American University of Beirut, Beirut, Lebanon) and stored at −20 °C. Venom was dissolved in E3 medium or ultrapure water prior to the experiments as a stock solution of 10 mg/mL.

### 2.2. Animal Handling and Ethics

Adult zebrafish were maintained at 28 °C in a 14/10 hours (hrs) light/dark cycle at the Lebanese University laboratories according to established procedures. The wild-type AB strain was used in this study. Maintenance of zebrafish stocks and experiments on larvae were carried out in accordance with the *Guidelines on the protection of experimental animals*, by the Council of Europe, which allow for the usage of zebrafish embryos up to 5 days after fertilization (approximately to the moment of independent feeding) without the need of a license. Zebrafish embryos were collected and allowed to develop at 28 °C. To prevent pigment formation, 0.2 mM phenylthiourea (PTU) was added to the fish water starting at 24 hrs postfertilization (hpf).

The C57BL/6 mice were fed a standard diet and kept at 25 °C in a 12 hrs day/night cycle. Animal care and use for this study were performed in accordance with the European Directive 2010/63/EU “On the protection of animals used for scientific purposes” and complied with the guidelines published in the *NIH Guide for the Care and Use of Laboratory Animals*. Euthanasia was performed using cervical dislocation.

### 2.3. Developmental Toxicity Assay

At 2 hpf, embryos were placed in 12-well plates with 1 mL of E3 medium and treated with venom at final concentrations of 1, 10^−1^, 10^−2^, or 10^−3^ mg/mL. Embryos treated with E3 medium were considered as negative controls, while embryos treated with retinoic acid at a final concentration of 0.5 µM served as positive controls on the teratogenic effects and associated lethality. Embryos were kept at 28 °C and checked for mortality rate and various types of developmental defects at different timepoints under a Carl Zeiss Stereomicroscope. The median lethal dose (LD_50_) value was calculated using the AAT Bioquest online tool [29].

### 2.4. Coiling Test

One-day-old embryos were manually dechorionated and placed in 24-well plates. Spontaneous tail coiling recordings were acquired using a Carl Zeiss Stereomicroscope. The video recording assembly was placed in a room maintained at a temperature of 28 °C. Spontaneous tail coiling videos were captured for 5 min, starting with 30 s of baseline recording before adding venom at a final concentration of 1 mg/mL. Embryos treated with E3 medium were considered as controls. Side-to-side movements of the tail were counted manually, and the quantification of spontaneous tail movement was expressed as coiling frequency (Hz).

### 2.5. Cell Culture and Differentiation

The P19 mouse embryonic carcinoma cells were differentiated into neurons using all-trans retinoic acid (ATRA) as described previously [30]. Briefly, P19 cells were maintained in T-75 culture flasks using low glucose (1 g/L) Dulbecco’s modified Eagle’s medium (DMEM) supplemented with 5% fetal calf serum (FCS), 5% delipidated FCS, and gentamycin (10 μg/mL). To induce differentiation, cells were washed twice with phosphate-buffered saline (PBS) and detached with 0.001% trypsin for 1 min. After trypsin neutralization, cells were centrifuged at 100 RPM for 5 min at room temperature (RT). Cell pellet was suspended in differentiation media (DM) consisting of α-MEM 1900 (Gibco) supplemented with 10% FCS. Next, 3 × 10^6^ cells were seeded in P10 Petri dishes containing DM supplemented with ATRA (5 µM) and allowed to form aggregates for 4 days at 37 °C in a humidified environment with 5% CO_2_. After 4 days, aggregates were collected by sedimentation, washed in PBS, and trypsinized with 0.25% trypsin for 3 min in a 37 °C water bath. After trypsin neutralization, cells were then mechanically dissociated by pipetting and filtered through a Corning^®^ cell strainer 40 µm nylon filter (Merck). After centrifugation, cells were suspended in Neuronal Medium (NM) consisting of DMEM (4.5 g/L glucose)-GLUTAMAX-1-Ham-F12 (1:1) medium supplemented with N2 (Gibco, 17502048) and 50 µM fibronectin (Merck), then seeded in 96-well plates precoated with 0.01% poly-L-lysine (Merck) at a density of 2 × 10^4^ per well. Half of the medium was changed every third day. Cells were allowed to differentiate for 7 days at 37 °C in a humidified environment with 5% CO_2_.

### 2.6. Primary Cortical Neuron Cultures

Primary cortical neuron cultures were prepared as described in [31]. Briefly, dissected cortices of E13.5 C57BL/6 mice were incubated in 150 µL of prewarmed trypsin/EDTA (Gibco, 25300-054) at 37 °C for 15 min with agitation (600 RPM). Dissociation was stopped by adding 850 µL of culture media consisting of neurobasal medium supplemented with 0.5 mM GlutaMAX, 1% penicillin/streptomycin, and 1% B-27 supplement. The tissue was then dissociated by gentle titration through a P1000 micropipette (15 times) and then a P200 micropipette (5 times) to obtain a single cell suspension. Cells were counted and seeded in 96-well plates precoated with 0.01% poly-L-lysine (Merck) at a density of 2 × 10^4^ per well. Half of the medium was changed every third day. Neurons were kept in culture, and analysis was performed after 7 days (DIV 7).

### 2.7. LDH Cytotoxicity Test

The Lactate Dehydrogenase (LDH) Cytotoxicity Assay Kit (Thermofisher) was used to measure the LDH released from the cytosol of lysed cells into the culture supernatants. Briefly, seeded neurons in 96-well plates were treated with different concentrations of snake venom (10^−1^, 10^−2^, and 10^−3^ mg/mL). Water and Triton (10%) were used as negative and positive controls, respectively. After incubating the plates at 37 °C, 5% CO_2_ for 4 hrs, 50 µL of the supernatant was transferred to 96-well plates and mixed with 50 µL of the reaction mixture. The plate was incubated for 30 min at RT protected from light. Then, the reaction was stopped with 50 µL of stop solution, and the absorbance was measured at 490 and 680 nm. Cytotoxicity percentages were calculated following the manufacturer’s recommendations.

### 2.8. Determination of Cardiovascular Toxicity and Hemorrhage

Two-day-old embryos were placed in 12-well plates with 1 mL of E3 medium and treated with the venom at final concentrations of 1 or 10^−1^ mg/mL. Embryos treated with E3 medium were considered as the negative control.

Heart rate was determined from videos taken under a Carl Zeiss Stereomicroscope after 10 and 30 min of the treatment and expressed as beats per minute (bpm). Embryos were also observed for any sign of hemorrhage under a Carl Zeiss Stereomicroscope after 6 hrs of incubation with the venom/E3 medium.

### 2.9. Statistical Analysis

Differences among groups were analyzed with GraphPad Prism 6.0 software (GraphPad Software Inc., San Diego, CA, USA) using the Student’s *t*-test. The results are expressed as the mean ± SEM.

## 3. Results

### 3.1. Montivipera bornmuelleri’s Venom Developmental Toxicity on Zebrafish Embryos

**Figure 1:**
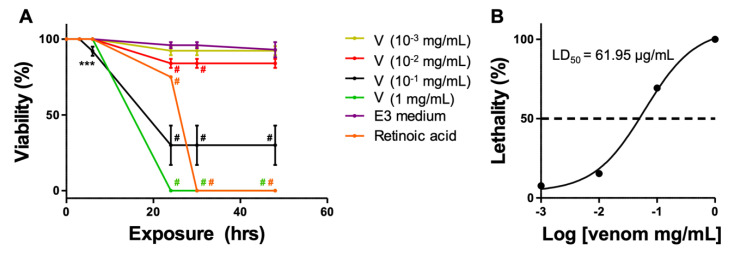
***M. bornmuelleri*’s venom toxicity on zebrafish embryos**. (**A**) Viability rates of zebrafish embryos exposed to different concentrations of the venom (V) in the early blastula stage (2 hpf). E3 medium was used as the negative control, while retinoic acid was used as the positive control. *** *p* ≤ 0.001 and # *p* ≤ 0.0001 compared to the negative control of each time point and color-coded for clarity. N = 13 for each venom condition, N = 30 for E3 condition, and N = 8 for retinoic acid condition. (**B**) Dose–response mortality curve of *M. bornmuelleri*’s venom on zebrafish embryos after 24 hrs of exposure. The LD_50_ value is indicated.

Zebrafish embryos at early blastula stage (2 hpf) were exposed to increased concentrations of *Montivipera bornmuelleri*’s venom and checked for mortality rate and various types of developmental defects during 48 hrs of exposure. The venom showed dose-dependent toxicity on zebrafish embryos (Figure 1A). In fact, all embryos were found dead after 24 hrs of exposure at 1 mg/mL. The percentage of viability increased when embryos were exposed to lower venom concentrations (33.3 ± 13% for 10^−1^ mg/mL; 85 ± 3% for 10^−2^ mg/mL; 91.7 ± 3% for 10^−3^ mg/mL). Interestingly, the fully penetrant lethality of the venom observed at 1 mg/mL was even more effective than retinoic acid (75% of viability), known for its teratogenicity and toxic effect; therefore, used as a positive control. These findings emphasize the toxicity of the *M. bornmuelleri*’s venom on zebrafish embryos. The toxicity observed at 24 hrs remained stable at 30 and 48 hrs; however, retinoic acid toxicity kept increasing in time (75% of viability at 24 hrs vs. 0% of viability at 30 hrs). Based on these findings, we estimated the venom’s LD_50_ at 61.955 µg/mL after 24 hrs of exposure (Figure 1B).

**Figure 2:**
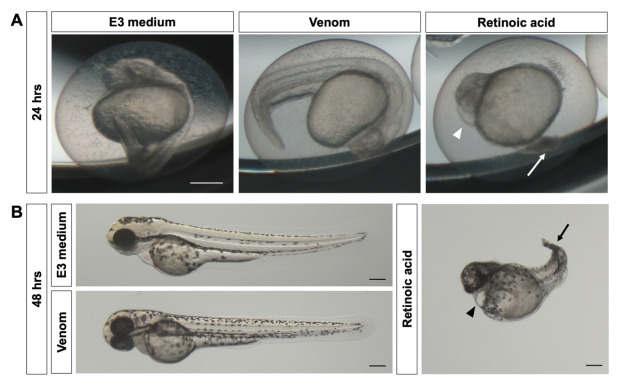
**Lack of teratogenicity of *M. bornmuelleri*’s venom on zebrafish embryos.** Representative images of zebrafish embryos after (**A**) 24 and (**B**) 48 hrs of exposure to *M. bornmuelleri*’s venom (1 mg/mL) showing no signs of teratogenicity. Embryos exposed to retinoic acid served as the positive control, showing signs of teratogenicity such as pericardial edema (arrowheads) and tail malformation (arrows). E3 medium was used as the negative control. Scale bar: 200 µm.

To check for possible teratogenic effects for the chosen venom concentrations, embryos were inspected under a microscope for morphological abnormalities throughout the experiment. Contrary to retinoic acid, known for its teratogenicity, the venom did not show any teratogenic effect during zebrafish embryo development neither at 24 nor 48 hrs after exposure (Figure 2A,B). In fact, embryos exposed to the highest concentration of venom (1 mg/mL) were phenotypically comparable to E3-treated (negative controls) but not to retinoic acid-treated (positive controls) embryos. Phenotypic observations show that venom-treated embryos developed normally without any sign of spinal curvature, pericardial edema, and tail malformation, contrary to retinoic acid-treated embryos that displayed these signs of teratogenicity (Figure 2A,B). Together these findings suggest that the zebrafish embryos’ death after being exposed to the venom was due to developmental toxicity rather than teratogenic effect.

### 3.2. Neurotoxicity of M. bornmuelleri’s Venom on Zebrafish Embryos

**Figure 3:**
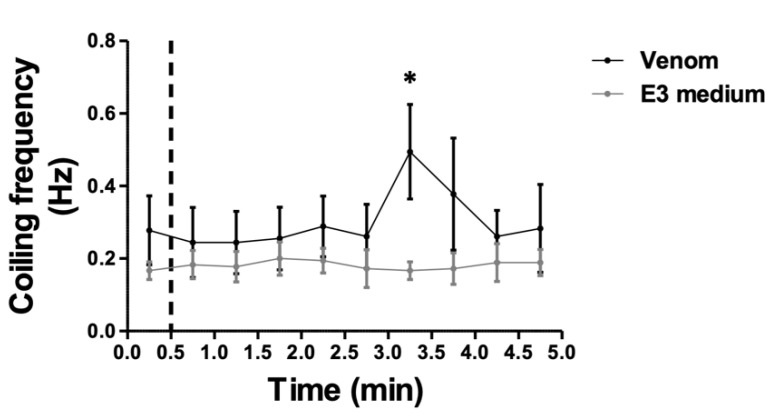
**Neurotoxicity of *M. bornmuelleri’s* venom**. The effect of *M. bornmuelleri*’s venom (1 mg/mL) on one-day-old embryos’ tail coiling frequency. Baseline coiling activity was recorded for 30 s before addition of the venom (dashed line). E3 medium was used as the negative control. * *p* ≤ 0.05; N = 6 per condition.

Analysis of early tail coiling activity of zebrafish embryos emerged recently as a powerful tool for neurotoxic compounds screening [24]. Therefore, we assessed spontaneous tail coiling of zebrafish embryos before and after their exposure to *M. bornmuelleri*’s venom (Figure 3). Baseline tail coiling frequencies were comparable to those studying the development of motor behaviors in the zebrafish embryos [28]. A significant increase in tail coiling frequency was observed 2.5 min after venom exposure (0.49 ± 0.13 Hz); however, tail coiling frequency remained stable in control embryos, and no significant increase was observed after E3 medium addition (0.16 ± 0.02 Hz). These findings show that *M. bornmuelleri*’s venom potentiates spontaneous tail coiling of zebrafish embryos, suggesting a neurotoxic effect.

**Figure A1:**
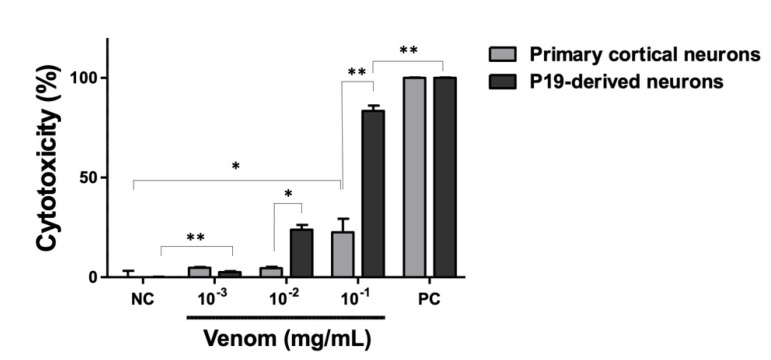
**Neurotoxic activity of *M. bornmuelleri*’s venom on primary cortical and P19-derived neurons.** Water and Triton (10%) were used as the negative (NC) and positive (PC) controls, respectively. * *p* ≤ 0.05; ** *p* ≤ 0.01.

To investigate this hypothesis, the neurotoxicity of *M. bornmuelleri*’s venom was also checked in vitro on P19-derived and primary cortical neurons. Results show dose-dependent toxicity of the venom on the two types of neuronal cells (Figure A1). Interestingly, neurons derived from P19 cells appeared to be more sensitive to the venom than primary cortical neurons, suggesting a selective toxicity that could be dependent on the neuron phenotype. In fact, for P19-derived neuronal cultures, significant toxicity appeared even with the lowest concentration of venom (10^−3^ mg/mL), which was not the case for primary cortical neurons (Figure A1). In addition, at the higher venom concentrations, the toxicity percentage was higher in P19-derived neuronal cultures than in the primary cortical cultures (23.84 ± 2.4% vs. 4.54 ± 0.7% and 83.44 ± 2.6% vs. 22.51 ± 6.8%, at 10^−2^ and 10^−1^ mg/mL, respectively). Taken together, our data describe a neurotoxic effect of *M. bornmuelleri*’s venom.

### 3.3. Cardiotoxic Effects of M. bornmuelleri’s Venom on Zebrafish Embryos

**Figure 4:**
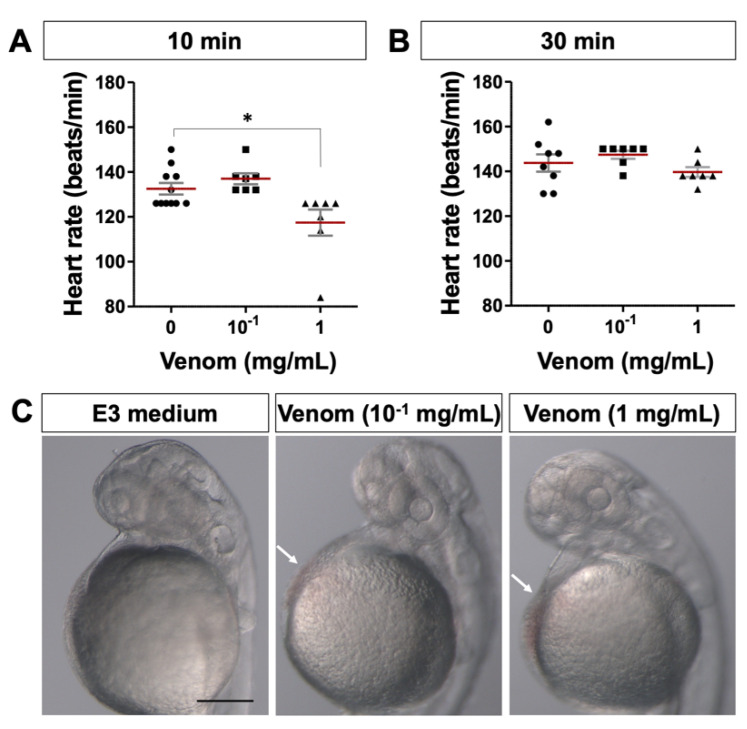
**Cardiotoxic effects of *M. bornmuelleri*’s venom on zebrafish embryos**. Heart rate (beats/min) of 2-day-old zebrafish embryos measured after 10 min (**A**) and 30 min (**B**) of venom addition. E3 medium was used as the negative control. * *p* ≤ 0.05; at least six embryos were analyzed per condition. (**C**) Representative images of 2-day-old embryos showing dose-dependent hemorrhage (white arrows) 6 hrs after venom addition. E3 medium was used as the negative control, showing no sign of hemorrhage. Scale bar: 200 µm.

The analysis of zebrafish embryo heart rate showed a significant decrease in heartbeat rate 10 min postexposure to 1 mg/mL of the venom (venom: 117.4 ± 5.8 bpm vs. control: 132.5 ± 2.6 bpm) (Figure 4A). This decrease disappeared and the heart rate recovered to values comparable to the negative control after 20 min (venom: 139.7 ± 2.1 bpm vs. control: 143.8 ± 3.8 bpm) (Figure 4B). However, exposure to a lower amount of venom (10^−1^ mg/mL) did not show any significant changes in heartbeat rate neither after 10 nor 30 min of exposure (Figure 4A,B). These results show that *M. bornmuelleri*’s venom led to cardiac rhythmic disturbances, which may be due to direct cardiotoxic effects.

In addition to heartbeat rate disturbances, we inspected the embryos for any sign of hemorrhage. Microscopic observations showed that, contrary to the control condition, zebrafish exposed to the venom presented redness in the pericardial region (Figure 4C). These findings suggest that the venom may be impairing vascular integrity leading to hemorrhage. Together, our data suggest that *M. bornmuelleri* venom components can target the cardiovascular system.

## 4. Discussion

Various biological activities of *Montivipera bornmuelleri* snake venom have been reported [2]; however, its activity on the nervous system has not yet been studied. In this study, we investigated the neurotoxicity of *M. bornmuelleri*’s venom using zebrafish, a predictive animal model for neurotoxicity screening [17,32]. We also used this in vivo model to highlight the ability of *M. bornmuelleri*’s venom to target the cardiovascular system.

Since this is the first study investigating the effects of *M. bornmuelleri*’s venom on zebrafish, it was important to start by checking for lethality and developmental impacts of the venom on zebrafish embryos. Interestingly, the venom did not show any teratogenic effects during zebrafish embryonic development; however, the venom exhibited a remarkable toxicity with an estimated LD_50_ of 61.955 µg/mL.

The neurotoxicity was first studied using a functional assay evaluating changes in spontaneous tail coiling in zebrafish embryos exposed to the venom. Our findings show that *M. bornmuelleri*’s venom potentiated spontaneous zebrafish coiling, highlighting the neurotoxicity of the venom. In fact, this strategy using zebrafish as a systems toxicology model emerged recently and is promising for developmental neurotoxicity screening [22,23,24,25]. During the early stages of zebrafish development, the advanced development of sensory-motor neuronal circuits translates into spontaneous side-to-side contractions of zebrafish trunk, the earliest motor behavior of the embryos [26,27,28]. This behavior is due to the neurotransmitters produced and released at early stages of zebrafish development [33], which can be targeted by toxicological compounds leading to an imbalance in neurotransmitters and the failure of central nervous system function. The exact target of *M. bornmuelleri*’s venom leading to spontaneous coiling potentiation is unknown and needs further investigation. However, one scenario can be suggested in which *M. bornmuelleri*’s venom interferes with cholinergic signaling. In fact, cholinergic neurons are well reported in one-day-old zebrafish embryos [34], and proper cholinergic signaling is important for spontaneous zebrafish coiling [35]. As such, *bajan* mutants, resulting from a point mutation in the gene coding choline acetyltransferase, which is the enzyme responsible for acetylcholine synthesis, lack the coiling movement [35]. Since *M. bornmuelleri*’s venom was able to potentiate spontaneous zebrafish coiling, it could be possible that the venom interferes with the cholinergic signaling pathway. For example, it has previously been shown that the inhibition of acetylcholinesterase leads to neuronal stimulation and hyperactivates spontaneous tail coiling [36]. Although inhibitors of acetylcholinesterase are known to be present in snake venom [37,38], other mechanisms could take place, especially as neuromuscular junctions have different sites of action of snake neurotoxins [39].

The suggested neurotoxic effect of the venom was also validated on neuronal cultures. In fact, *M. bornmuelleri*’s venom was cytotoxic on neuronal-like cultures (P19-derived neurons) and primary cortical neurons. Interestingly, P19-derived neuronal cultures were more sensitive to the venom compared to primary cortical cultures, which might be due to the phenotype of the neuronal culture. Primary cortical neurons are GABAergic and glutamatergic [40,41,42], expressing gamma-aminobutyric acid (GABA) and glutamate receptors [43,44,45]. P19-derived neuronal cultures are striatal-like GABAergic neurons [30], showing spontaneous neuronal network activity within 6 days [46]. These neurons are responsive to GABA and glutamate neurotransmitters, and they highly express GABA receptors [46]. Overactivation of GABA receptors has been shown to be neurotoxic, while chronic blockade of these receptors enhances neurons’ survival [47]. Therefore, it could be possible that *M. bornmuelleri*’s venom potentiates the GABAergic system, leading to neuronal death. Since glutamatergic toxicity is also well reported [43], another scenario that can be proposed is that the venom induces neuronal death by acting on glutamate receptors, leading to cytotoxic intracellular Ca^2+^ overload. Overall, the exact mechanism in which venom’s neurotoxicity occurs needs further investigation.

P19-derived neuronal cultures also express the dopamine D2 receptor, a molecular characteristic of striatopallidal medium spiny neurons (MSNs) [30]. MSNs represent 95% of the neuronal population in the striatum [48], a critical hub for locomotion control [49]. The high toxicity of the venom on this neuronal model could therefore suggest the observed motor behavior defects in a recently reported case of *M. bornmuelleri* snake bite [50]. In addition to motor behavior impairments, the envenomated patient showed other neurological dysfunction, highlighting the neurotoxicity of *M. bornmuelleri*’s venom. Together these findings encourage the in-depth exploration of the venom’s components that induce neurotoxicity, probably by modulating ion channels.

Moreover, the venom induced vascular damage in zebrafish embryos leading to hemorrhage. This might be due to the metalloproteinases in *M. bornmuelleri*’s venom [3] and be responsible for the hemorrhagic activity [51]. In addition, the venom induced negative chronotropic effects reducing heartbeat rate. Since vasodilation leads to an immediate decrease in blood pressure and heart rate [52], our findings correlate with previous ex vivo studies describing the vasorelaxant effect of *M. bornmuelleri*’s venom [7,12]. Together, these findings suggest that the venom could act on the cardiovascular system by altering vascular integrity and inducing cardiac arrythmia. This might be due to the presence within the venom of a cocktail of compounds that targets the cardiovascular system.

## 5. Conclusions

In this study, we highlighted the neurotoxicity of *M. bornmuelleri* snake venom using both zebrafish and in vitro neuronal culture models. Our data showed that the venom also targets the cardiovascular system, highlighting a broad spectrum of biological activities. Despite the advantages of cell culture techniques, such as consistency and reproducibility, zebrafish can be considered a powerful tool for venom research studies, as it represents a complex developing organism.

## Data Availability

The data presented in this study are available upon request from the corresponding author.
